# Conductive Membranes Based on Cotton Fabric Coated with Polymers for Electrode Applications

**DOI:** 10.3390/ma15207286

**Published:** 2022-10-18

**Authors:** Raluca Maria Aileni, Laura Chiriac

**Affiliations:** Department of Research in Textile Materials Engineering and Processes, National Research and Development Institute for Textiles and Leather, 030508 Bucharest, Romania

**Keywords:** electrodes, sensors, textile, cotton, conductive

## Abstract

This paper presents the evaluation of some electrodes based on polymeric conductive membranes (polyvinylidene fluoride (PVDF), polyvinyl alcohol (PVA) and polyethylene glycol (PEG)) for sensor applications. The electrodes were developed using textile support (weave structure-based 100% cotton yarns) and applying conductive membrane layers deposited on the textile surface. Coating the fabrics with thin layers of conductive membranes could generate new surfaces with the electrical resistance specific to conductive samples. Laboratory tests evaluated the physicomechanical and electrical properties. The surface resistance was investigated using a digital surface resistance meter by neglecting electrode polarization impedance. In addition, the correlation coefficients between the physicomechanical and electrical parameters obtained by the laboratory were analyzed. These conductive samples can be used to and develop flexible electrodes for moisture, temperature and strain sensors.

## 1. Introduction

Due to the miniaturization of the electronic components using advanced assembly technologies and flexible support, printed electronics, and the wearable devices-based sensors having self-powering capacity and autonomy represent a challenge for many researchers. Users mainly prefer wearable devices (smart-glass, intelligent clothes, intelligent rings) because they are flexible, lightweight and comfortable. The scientific articles reported using polyvinylidene fluoride (PVDF) membranes and nanofibres for energy harvesting devices [1,2,3].

PVDF membranes [4,5] have been used often for intelligent textile, capacitive sensors for humidity detection, triboelectric textile sensors and energy harvesters [6,7,8,9].

Research shows that piezoelectric wearable sensors for biomedical signals monitoring is efficient because using PVDF can obtain flexible membranes that are biocompatible and lightweight [10]. Mix PVDF with different microparticles or cellulose nanocrystal to obtain nanocomposite fibers by dry-jet wet spinning [11].

Overall, the PVDF membranes are also used for triboelectric nanogenerator (TENG) [12,13,14,15] or self-powered sensors [16,17,18,19,20] for pressure or vibration measurement [21,22,23,24] based on the electrical properties of the PVDF polymers [25,26,27,28].

The purpose of this research was to develop wearable electrodes-based polymeric membranes (PVDF), polyvinyl alcohol (PVA), or polyethylene glycol (PEG) for sensors using textile support coated with conductive paste-based polymeric matrix and microparticles (copper, nickel, graphene oxide).

Moreover, the conductive composites based on graphene oxide [29,30,31,32,33,34] and polyaniline [35] have been used to develop strain or biomedical sensors and energy harvesting.

## 2. Materials and Methods

To achieve the proposed results consisting of conductive polymeric membrane electrodes, in our experiments, the textile samples (plain weave based 100% cotton S twisted yarns Nm 20/2 (FAVIL SA, Râmnicu Vâlcea, Romania), breaking strength 1758 cN, breaking elongation 8.46%) were treated with conductive paste based polymeric matrix: polyvinyl alcohol (PVA (Sigma Aldrich, Taufkirchen, Germany)), PVDF (Sigma Aldrich, Taufkirchen, Germany), polyethylene glycol (PEG (Sigma Aldrich, Taufkirchen, Germany)), polyvinylpyrrolidone (PVP (Sigma Aldrich, Taufkirchen, Germany)), distilled water (dH_2_O), dimethylformamide (DMF) 99.5+%, pure and microparticles (copper (Cu_1_ (Sigma Aldrich, Taufkirchen, Germany) having particle dimensions between 14–25 µm; Cu_2_ (Sigma Aldrich, Taufkirchen, Germany) having particle dimensions <75 µm), nickel (Ni (Sigma Aldrich, Taufkirchen, Germany) having particle dimensions of 150 µm), and graphene oxide (GO)). All these samples were treated previously using a primary classical cleaning method (alkaline boiling-bleaching) to obtain a hydrophilic character of the surface.

The samples (electrode materials) have been prepared by coating the textile surface with a conductive paste-based polymeric matrix (PVA, PEG, PVP, and PVDF) with microparticles content (GO, Cu_1_, Cu_2_, Ni) using the scrapping method.

The polymers such as PEG, PVA, and PVP present a hydrophilic character. The polymer PVDF has a nonpolar phase and is hydrophobic. In order to transform hydrophobic PVDF into hydrophilic, the nonpolar PVDF polymer has been dissolved in polar (hydrophilic) aprotic solvent DMF. The polymeric matrix PVDF with microparticle content presents a hydrophilic surface.

According to the standard SR EN ISO 9237:1999, the air permeability (Pa) of the electrode materials obtained was investigated using a Testex Air Permeability Tester FX 3300 (TEXTEST AG, Schwerzenbach, Switzerland).

The contact angle was investigated using the Video Camera Angle (VCA) Optima device (AST Products, Inc., Billerica, MA, USA), which has a CCD camera, syringe with distilled water, and droplet size of 4 µL.

**Figure 1:**
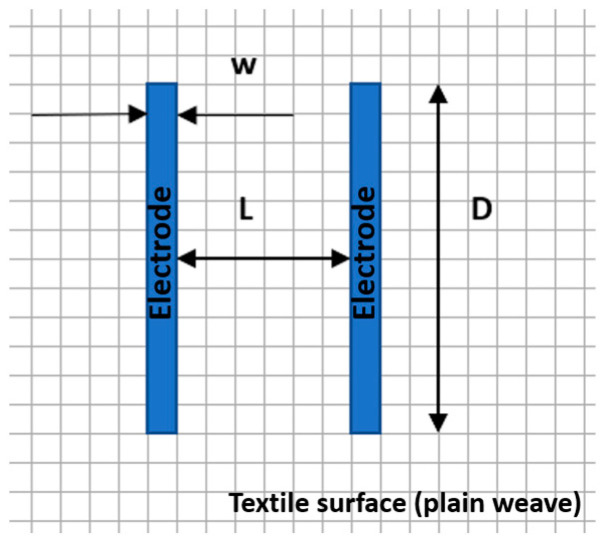
Placement of the parallel electrodes on the same side of the textile surface for surface resistance measurement (w represents the width of the electrode, D represents the length of the electrode (D = 6 cm), and L is the distance between electrodes (L = 3 cm)).

The electrical resistance (Rs) has been investigated using portable resistance meter devices with internal parallel electrodes (Figure 1) fixed on the textile surface having 6 cm length (D), 0.5 cm width (w) and 3 cm distance (L) between the parallel electrodes. The tests neglected the polarization phenomena on the electrodes.

The surface morphology was evaluated using scanning electron microscopy (Quanta 200 184 SEM) and the surface topography using a digital optic microscope (Biolux, Bresser, Germany).

Table 1 presented:

- the composition of samples 1–14 is based on the polymeric matrix (PVDF (samples 2–5), PEG (samples 6–10 and 14), PVP (samples 5–12 and 13–14), and PVA (sample 6)) and microparticles (GO (samples 2 and 9), Ni (samples 3 and 11), Cu_1_ (samples 4, 8, 10, 12 and 14) and Cu_2_ (samples 5–7 and 13));
- the results of the physico-mechanical (Mass per unit area (SR EN 12127:2003), thickness (SR EN ISO 5084:2001), air permeability (SR EN ISO 9237:1999), water vapor permeability (STAS 9005:1979)) and electrical resistance (Rs) evaluations.

The samples based on PVDF polymer matrix and Cu_1_, Cu_2,_ GO and Ni microparticles were treated by immersion in artificial perspiration solutions (alkaline/acid) for 10 min. The surface electrical resistances were analyzed, and it can be concluded that the treatment in acid/alkaline perspiration solutions leads to an antistatic character of textile surfaces (10^9^ Ω). Table 2 presents the electrical resistance values for samples based PVDF polymeric matrix. For testing, five samples were used for each paste composition with dimensions 10 × 10 cm.

In Table 2, it can be observed that samples T1–T4, which are samples 2–5 from Table 1 treated with a conductive paste based:

- PVDF and Cu_1_ microparticles (T3), the surface resistance decreases by 10%;
- PVDF and Cu_2_ microparticles (T4), the surface resistance has the same value of 10^9^ Ω;
- PVDF and Ni microparticles (T2), the surface resistance increases by 200%;
- PVDF and GO microparticles (T1), the surface resistance decreases by 25%.

The alkaline perspiration solution contains:

- distilled water;
- l-histidine monohydrochloride monohydrate;
- sodium chloride;
- disodium hydrogen orthophosphate dodecahydrate;
- disodium hydrogen orthophosphate dihydrate.

The acid perspiration solution contains:

- distilled water;
- l-histidine monohydrochloride monohydrate-
- sodium chloride;
- sodium dihydrogen orthophosphate dihydrate (ISO 105-E04:2013).

We observed that alkaline/acid perspiration treatments generate an antistatic character calibration of the surface resistance. In addition, this calibration was also observed in our previous experiments based on coating textile with an electroconductive paste based on chitosan. From Table 1, sample 3 (based on PVDF and Ni microparticles) presents the adequate surface resistance specific to electroconductive materials and can be used for electrode manufacturing. In addition, samples 5–10 and 14 present the resistance values specific to dissipative materials, while samples 2, 4 and 11–13 present a surface resistance specific to materials used for electrical insulation.

## 3. Results

### 3.1. Characterization of the Samples Treated Using Conductive Membranes

#### 3.1.1. Surface Morphology Using SEM

A scan electron microscopy (SEM) using Quanta 200 184 SEM (FEI, Cleveland, OH, USA) was used to study the surface morphology using 2000× magnification.

The surface morphology of the fabrics treated using polymeric (PVDF) paste (samples 2–5) with metallic and GO microparticles content is presented in Table 3. Sample 1 from Table 3 represents the hydrophilic fabric (100% cotton, plain weave) used to obtain samples 2–14.

Table 3 shows the morphology of the textile surface untreated (sample 1) and coated with conductive paste-based graphene (sample 2), Ni (sample 3), Cu_1_ (sample 4) and Cu_2_ (sample 5). From the surface morphology of sample 4, in comparison with sample 5, it can be observed that the Cu_1_ particles (14–25 µm) have a homogeneous distribution on the textile surface. In the case of sample 3 based on Ni microparticles, a tendency of microparticles to agglomerate and to form clusters of microparticles can be observed. In addition, samples 2 (based on graphene) and 5 (based on Cu2 microparticles having dimensions <75 µm) present a non-homogeneous distribution of the particles.

In the case of samples 2, 3, 4 and 5 (Table 3), it can be observed that the aggregations of the microparticles are reduced after alkaline and acid perspiration treatments. This is because the PVA, PEG, and PVP are hydrophilic polymers, and PVDF dissolved in the organic solvent DMF becomes hydrophilic. Consequently, the sample surface becomes hydrophilic after coating through thin film deposition using screen printing the conductive paste on the textile surface. This explains that some metal microparticles may migrate in the water-based alkaline/acid perspiration solution after the immersion of the samples in acid/alkaline perspiration for 10 min. In addition, we observed that, after the treatments with alkaline and acid artificial perspiration, the electrical resistance (Rs) was reduced by 25% for GO-based samples, by 10% for Cu_1_-based samples and was increased by 200% in the samples based on Ni microparticles.

#### 3.1.2. Surface Topography Using Optical Microscopy

Table 4 presents the electrodes’ topography captured by digital electron microscopy (60× magnification) for raw fabrics and fabrics coated (samples 2–5) with conductive paste-based polymeric matrix (PVDF, PVA, PEG) and microparticles (Cu_1_, Cu_2_, Ni, GO). In addition, Table 4 presents the static capture of the contact angles. We can observe the contact angles measured using the VCA Optima device (Figure 1) and a four μL distilled water droplet, with values greater than 90° for samples 2 and 4. However, for other samples (6–14, presented in Table S1, in the Supplementary Materials), the contact angles are equal to zero, indicating that the samples present a pronounced hydrophilic character after coating with conductive polymeric paste.

#### 3.1.3. Chemical Composition

**Figure 2:**
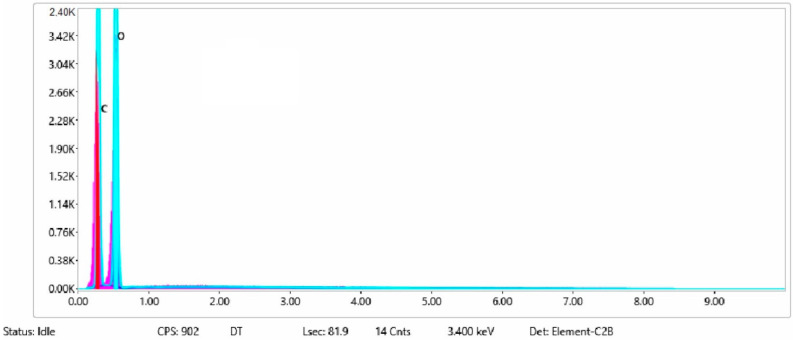
EDS spectra for sample 1 (fabric treated using a preliminary classical cleaning method, without conductive paste).

**Figure 3:**
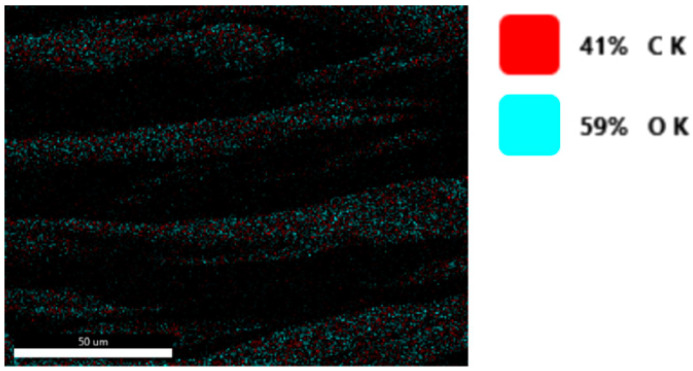
Smart elemental mapping overlay for sample 1 (fabric treated using a preliminary classical cleaning method, without conductive paste).

The chemical composition after coating was investigated using EDS (Energy Dispersive Spectroscopy) analysis and elemental mapping overlay for samples 1, 2, 3, 4 and 5 (Table 2) using EDAX (AMETEK, Inc., Montvale, NJ, USA), and the results are presented in Table 5. Using the smart phase mapping tool from EDAX, the spectra, elemental and phase maps were automatically collected for samples 2a, 2b, 2c, 3a, 3b, 3c, 4a, 4b, 4c, 5a, 5b and 5c. Samples 2, 3, 4 and 5 are treated with a polymeric conductive paste based on graphene (sample 2), nickel (sample 3), Cu_1_ (sample 4) and Cu_2_ (sample 5). Samples 2a, 3a, 4a and 5a are initially treated with polymeric conductive paste and after with artificial acid perspiration (samples 2b, 3b, 4b and 5b) and respective alkaline perspiration (2c, 3c, 4c and 5c). In Figure 2 and Figure 3 are presented the EDS spectra for sample 1 (clean fabric without conductive paste), respective the smart elemental mapping for sample 1.

**Figure 4:**
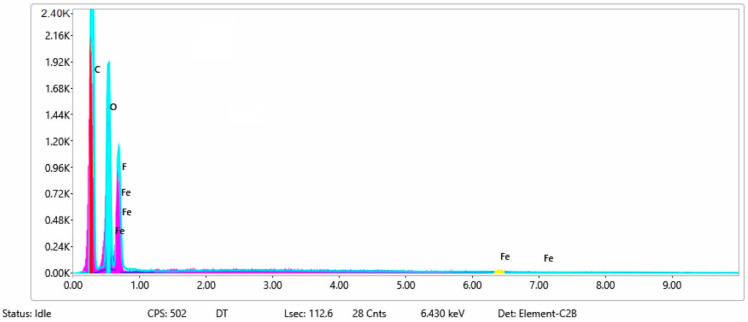
EDS spectra for sample 2 (fabric treated using a preliminary classical cleaning method and conductive paste).

**Figure 5:**
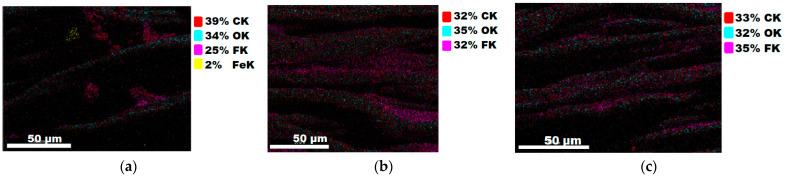
Smart elemental mapping overlay for sample 2: (**a**) untreated with artificial perspiration; (**b**) treated with acid perspiration; (**c**) treated with alkaline artificial perspiration.

Figure 4 and Figure 5 present the EDS spectra for sample 2 (clean fabric coated with conductive paste based PVDF and GO), respective the smart elemental mapping overlay for sample 2 untreated with artificial perspiration (Figure 5a), treated with acid perspiration (Figure 5b) and alkaline perspiration (Figure 5c).

**Figure 6:**
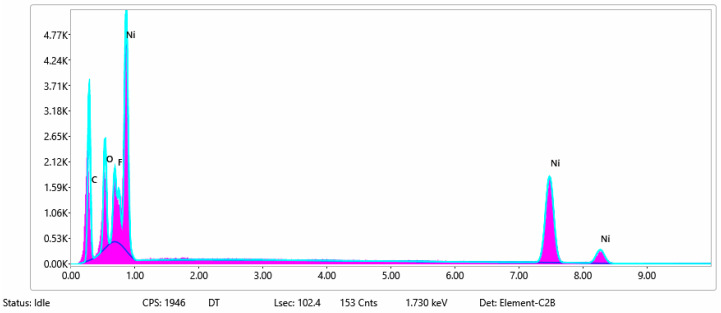
EDS spectra for sample 3 (fabric treated using a preliminary classical cleaning method and conductive paste).

**Figure 7:**
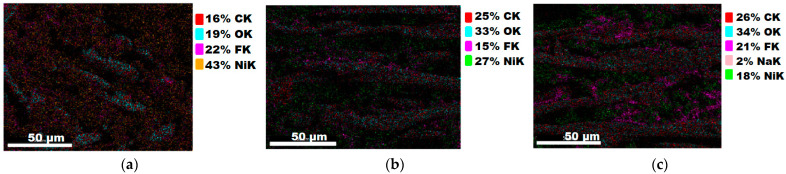
Smart elemental mapping overlay for sample 3: (**a**) untreated with artificial perspiration; (**b**) treated with acid perspiration; (**c**) treated with alkaline artificial perspiration.

In Figure 6 and Figure 7 are presented the ESD spectra for sample 3 (clean fabric coated with conductive paste based on PVDF and Ni, Figure 6), respective the smart elemental mapping for sample 3 untreated with artificial perspiration (Figure 7a), treated with acid perspiration (Figure 7b) and alkaline perspiration (Figure 7c).

**Figure 8:**
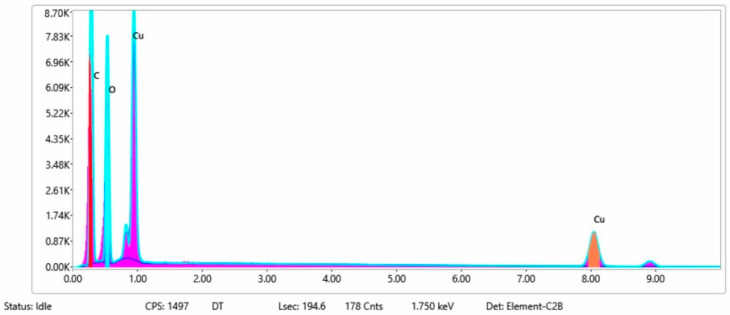
EDS spectra for sample 4 (fabric treated using a preliminary classical cleaning method and conductive paste).

**Figure 9:**
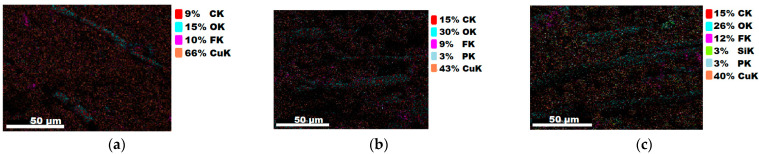
Smart elemental mapping overlay for sample 4: (**a**) untreated with artificial perspiration; (**b**) treated with acid perspiration; (**c**) treated with alkaline artificial perspiration.

In Figure 8 and Figure 9 are presented the ESD spectra for sample 4 (clean fabric coated with conductive paste based on PVDF and Cu_1_, Figure 8), respective the smart elemental mapping for sample 4 untreated with artificial perspiration (Figure 9a), treated with acid perspiration (Figure 9b) and alkaline perspiration (Figure 9c).

**Figure 10:**
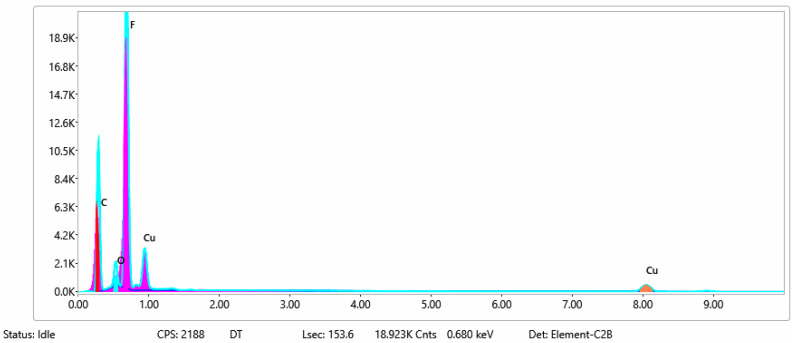
EDS spectra for sample 5 (fabric treated using preliminary classical cleaning method and conductive paste).

**Figure 11:**
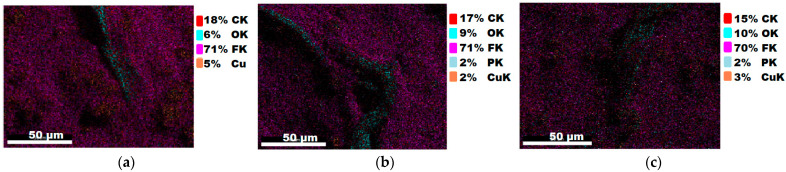
Smart elemental mapping overlay for sample 5: (**a**) untreated with artificial perspiration; (**b**) treated with acid perspiration; (**c**) treated with alkaline artificial perspiration.

In addition, Figure 10 and Figure 11 present the ESD spectra for sample 5 (clean fabric coated with conductive paste based on PVDF and Cu_2_, Figure 10), respective the smart elemental mapping for sample 5 untreated with artificial perspiration (Figure 11a), treated with acid perspiration (Figure 11b) and alkaline perspiration (Figure 11c). Overall, it can be observed that the quantity of the metals (Cu, Ni) is reduced after these treatments. For example, in the case of the sample:

- 3 (Figure 7a), the quantity of Ni was reduced by 11% after treatment with acid perspiration (Figure 7b), with a respective 25% after treatment with alkaline artificial perspiration (Figure 7c);
- 4 (Figure 9a), the quantity of Cu_1_ was reduced by 23% after treatment with acid perspiration (Figure 9b), with a respective 26% after treatment with alkaline artificial perspiration (Figure 9c);
- 5 (Figure 11a), the quantity of Cu_2_ was reduced by 3% after treatment with acid perspiration (Figure 11b), with a respective 2% after treatment with alkaline artificial perspiration (Figure 11c).

The reduction of the metal microparticles after treatments in acid or alkaline artificial perspiration explains the surface resistance increase in sample 3 coated with a paste-based Ni and PVDF.

## 4. Discussion

### 4.1. Correlation between Physicomechanical and Electrical Parameters

The correlation coefficients Pearson (1) between the dependent variable (surface resistance (*Rs*) after crosslinking) and independent variables (*Pa*, *Pv*, *δ* and *M*) have been analyzed to observe the strength of the relationship between these variables:

(1) $$r_{xy}=\frac{\frac{1}{n}\sum^{\;}\left(x-\bar{x}\right)\left(y-\bar{y}\right)}{s_{x}s_{y}}$$

where:

*x*, *y* represents the individual values of the variables *x* and *y*;

$\bar{x}$, $\bar{y}$ represents the arithmetic mean of all the values of *x*, *y*;

$s_{x},\;\;s_{y}$ represent the standard deviation of all values *x* and *y*.

**Figure 12:**
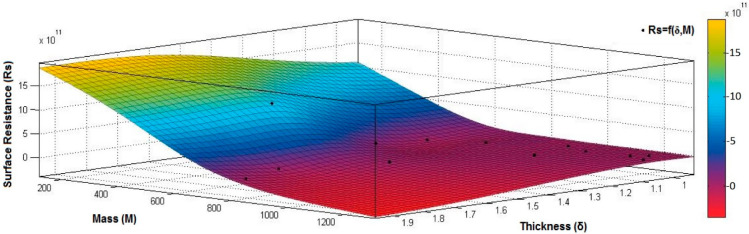
3D representation *Rs* = f(*M*, *δ*) samples coated with conductive polymeric paste.

**Figure 13:**
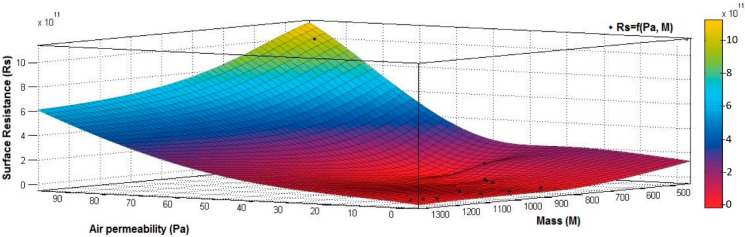
3D representation *R_s_* (*Pa*, *M*) samples coated with conductive polymeric paste.

**Figure 14:**
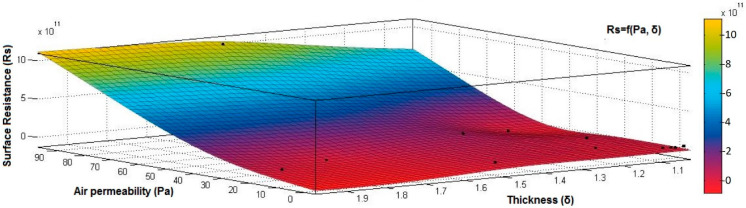
3D representation *Rs* = f(*Pa*, *δ*) samples coated with conductive polymeric paste.

Figure 12, Figure 13 and Figure 14 present the 3D interpolation of datasets for surface electrical resistance (*Rs*) values, mass (*M*) values, air permeability (*Pa*), and thickness (*δ*) values. These 3D interpolations (Figure 12, Figure 13 and Figure 14) were considered the independent variables such as mass (*M*), air permeability (*Pa*), thickness (*δ*) and surface resistance (*Rs*) as the dependent variables. Figure 12 presented the 3D representation of the multivariate dependence of *Rs* values on the mass and thickness values. Figure 13 presents the 3D representation of the *Rs* values dependent on mass and air permeability values. Figure 14 presents the 3D representation of the multivariate dependence of the electrical surface resistance (*Rs*) on the air permeability and thickness. The models from Figure 12, Figure 13 and Figure 14, using 3D multivariate representation, were developed to investigate and predict the *Rs* variable evolution depending on the independent variables such as mass, thickness, and air permeability.

Analyzing the correlation coefficient between *Rs* and *Pa* (*r_Rs_*_,*Pa*_ = 0.8523 (2)), we can observe a strong positive correlation between variables, which means that the increase of the *Pa* values can increase surface resistance values.

Analyzing the correlation coefficient between *Rs* and *δ* (*r_Rs,δ_ =* 0.2694 (3)), we can observe that, between *Rs* and *δ*, it is a weak correlation, and the increase or decrease of *δ* values will not generate modification in *Rs*.

Analyzing the correlation coefficient between *Rs* and *M* (*r_Rs_*_,*M*_ = −0.5261 (4)), we observed a weak negative correlation, and this indicates an inverse proportionality report between *Rs* and *M*, suggesting that the increase of *M* values can have a minor influence on the decrease of the *Rs* values:

(2) $$\;r_{Rs,Pa}=\left|\begin{array}{cc} 0.0000 & 0.8523\\ 0.8523 & 0.0000 \end{array}\right|⟺r12_{Rs,Pa}=r21_{Rs,Pa}=0.8523$$

(3) $$r_{Rs,\delta }=\left|\begin{array}{cc} 0.0000 & 0.2694\\ 0.2694 & 0.0000 \end{array}\right|⟺r12_{Rs,\delta }=r21_{Rs,\delta }=0.2694$$

(4) $$r_{Rs,M}=\left|\begin{array}{cc} 0.0000 & -0.5261\\ -0.5261 & 0.0000 \end{array}\right|⟺r12_{Rs,M}=r21_{Rs,M}=-0.5261$$

**Figure 15:**
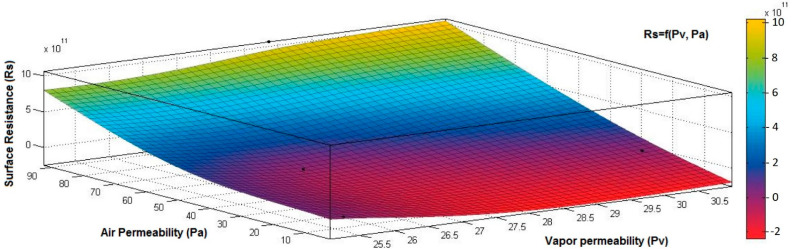
3D representation *Rs* = f(*Pa*, *Pv*) samples coated with a conductive polymeric paste-based PVDF.

**Figure 16:**
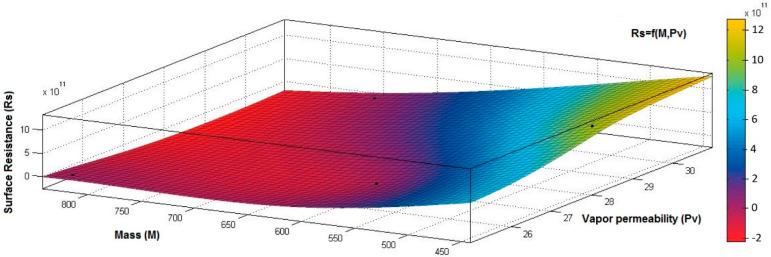
3D representation *Rs* = f(*M*, *Pv*) samples coated with a conductive polymeric paste-based PVDF.

**Figure 17:**
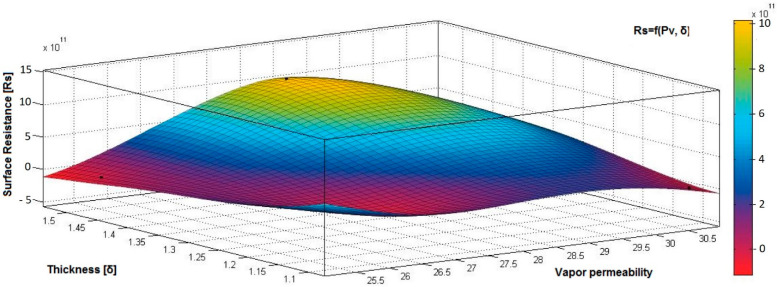
3D representation *Rs* = f(*δ*, *Pv*) samples coated with a conductive polymeric paste-based PVDF.

**Figure 18:**
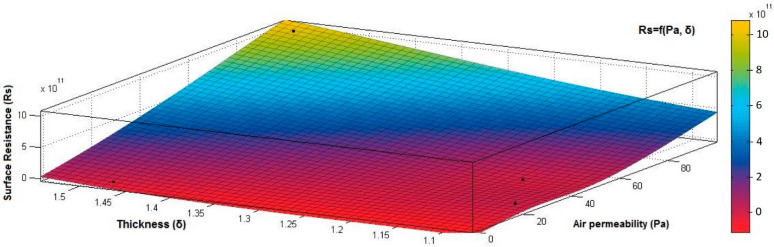
3D representation *Rs* = f(*δ*, *Pa*) samples coated with a conductive polymeric paste-based PVDF.

**Figure 19:**
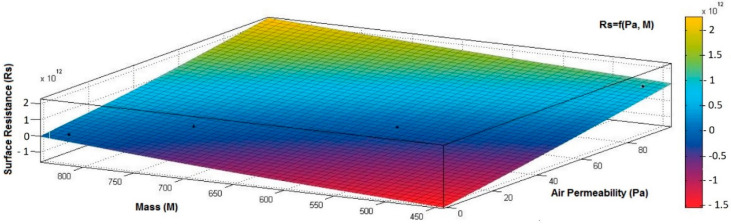
3D representation *Rs =* f*(M*, *Pa)* samples coated with a conductive polymeric paste-based PVDF.

Figure 15, Figure 16, Figure 17, Figure 18 and Figure 19 present the 3D representation of the surface resistance (*Rs*) depending on the independent variables air permeability (*Pa*), vapor permeability (*Pv*), mass (*M*) and thickness (*δ*) for electrodes based PVDF membranes. Figure 15 presents the 3D representation of *Rs* values dependent on air and vapor permeability. In Figure 16, the 3D representation of the *Rs* values dependent on mass and vapor permeability values is shown.

Figure 17 presents the 3D representation of the electrical surface resistance (*Rs*) depending on the thickness (*δ*) and vapor permeability (*Pv*).

Figure 18 presents the 3D representation of the electrical surface resistance (*Rs*) depending on the thickness (*δ*) and air permeability (*Pa*).

Figure 19 presents the 3D representation of the electrical surface resistance (*Rs*) depending on the mass (*M*) and air permeability (*Pa*).

In the case of the electrodes obtained by conductive PVDF membranes, the correlation coefficient values between surface resistance (*Rs*) and air permeability (*Pa*), thickness (*δ*), vapor permeability (*Pv*), and mass (*M*), we can observe the associations between variables analyzed.

The correlation coefficient between *Rs* and *Pa* (*r_Rs,Pa_* = 0.8969 (5)) is positive. It indicates a strong association between variables and means that the increase of the *Rs* can be influenced by increasing the value of *Pa*. The correlation coefficient between *Rs* and *δ* (*r_Rs,δ_* = 0.6392 (6)) is positive. It indicates a weak positive association between these variables, which means that the increase of *δ* will generate low *Rs* values.

The correlation coefficient between *Rs* and *Pv* (*r_Rs,Pv_* = 0.2082 (7)) is positive but below 0.3, indicating a low association between *Rs* and *Pv* variables. However, increasing the *Pv* value will not generate modification in *Rs*. In addition, the correlation coefficient between *Rs* and *M* (*r_Rs,M_* = −0.8046 (8)) is negative, suggesting a strong negative association between these variables and an inverse proportionality relationship:

(5) $$r_{Rs,Pa}=\left|\begin{array}{cc} 0.0000 & 0.8969\\ 0.8969 & 0.0000 \end{array}\right|⟺r12_{Rs,Pa}=r21_{Rs,Pa}=0.8969$$

(6) $$r_{Rs,\delta }=\left|\begin{array}{cc} 0.0000 & 0.6392\\ 0.6392 & 0.0000 \end{array}\right|⟺r12_{Rs,\delta }=r21_{Rs,\delta }=0.6392$$

(7) $$r_{Rs,Pv}=\left|\begin{array}{cc} 0.0000 & 0.2082\\ 0.2082 & 0.0000 \end{array}\right|⟺r12_{Rs,Pv}=r21_{Rs,Pv}=0.2082$$

(8) $$r_{Rs,M}=\left|\begin{array}{cc} 0.0000 & -0.8046\\ -0.8046 & 0.0000 \end{array}\right|⟺r12_{Rs,M}=r21_{Rs,M}=-0.8046$$

### 4.2. Electrodes Materials Comparison

Table 6 presents the resistance of the PVDF, PEG, PVP, and PVA-based sensors reported in the scientific literature of ethanol biosensors based on polymer-modified electrodes reported in the literature.

## 5. Conclusions

In conclusion, samples coated with a conductive polymeric paste-based PVDF, PEG, PVA and microparticles (Cu, Ni, and GO) present differences in surface resistance. Samples functionalized by PVDF with Ni microparticles content have a lower surface resistance (Rs = 10^3^ Ω) specific to the electroconductive materials. These conductive membranes obtained can be used for electrodes in flexible electronics.

In the case of the samples treated with artificial perspiration, a reduction of the surface resistance was observed with 25% for electrodes-based PVDF and GO, with a respective 10% for electrodes-based PVDF and Cu_1_ microparticles [50].

Analyzing the correlation parameters (surface resistance and mass, air permeability and vapor permeability) allows the identification of sensors that can be developed using the proposed electrodes.

In the case of the electrodes based on PVDF membrane:

- the correlation coefficient between electrical surface resistance (Rs) and vapor permeability (Pv) is 0.2082 (below 0.3), and this demonstrates that, between Rs and Pv, it is a weak correlation;
- the correlation coefficient between electrical surface resistance (Rs) and mass (M) is negative (−0.8046) and indicates a strong negative association between Rs and M, suggesting that increasing the M (quantity of conductive paste) will generate a decrease in the Rs;
- the correlation coefficient between Rs and Pa is positive (0.8969), indicating a strong positive association between variables and a direct relationship between Rs and Pa, suggesting that increasing the Pa (air being an excellent electrical insulator) will increase the Rs.

The proposed electrodes based on PVDF and Ni showed promising results compared to other state-of-the-art methods.

Our flexible electrodes generate new perspectives on the successful detection of temperature or moisture by integrating these electrodes in wearable textile articles.

In the future, we plan to use these electrodes based on PVDF and Ni in real demonstrators for moisture and temperature sensors.

Our next research objective is to develop a wearable sensor capable of detecting temperature and moisture variation by connecting the electrodes embedded in textile articles to a microcontroller.

## Figures and Tables

**Table 1:** Physico-mechanical and electrical properties of the electrodes-based polymeric membranes with metallic microparticles (Ni, Cu_1_, Cu_2_) and GO.

Sample No.	PVDF	PEG	PVP	PVA	GO	Ni	Cu_1_	Cu_2_	Rs * (Ω)	M ** (g/m^2^)	δ *** (mm)	Pv ****	Pa ***** (L/m^2^/s)
1	-	-	-	-	-	-	-	-	10^12^	415	1.06	-	33.76
2	x	-	-	-	x	-	-	-	10^12^	460.4	1.52	28.5	95.2
3	x	-	-	-	-	x	-	-	10^3^	593.6	1.144	26.7	45.9
4	x	-	-	-	-	-	x	-	10^10^	748	1.096	30.7	24.42
5	x	-	x	-	-	-	-	x	10^9^	824.4	1.474	25.3	3.332
6	-	x	x	x	-	-	-	x	10^6^	997.6	1.168	-	10.86
7	-	x	x	-	-	-	-	x	10^6^	1042	1.044	-	1.38
8	-	x	x	-	-	-	x	-	10^7^	1262	1.06	-	2.066
9	-	x	x	-	x	-	-	-	10^7^	940.4	1.008	-	2.076
10	-	x	x	-	-	-	x	-	10^7^	1222	1.006	-	2.024
11	-	-	x	-	-	x	-	-	10^10^	717.6	1.732	24.1	27.44
12	-	-	x	-	-	-	x	-	10^11^	608	1.322	24.1	36.74
13	-	-	x	-	-	-	-	x	10^10^	784.8	1.898	26	19.68
14	-	x	x	-	-	-	x	-	10^6^	1187.2	1.034	-	1.184

* Rs—surface resistance [Ω]; ** M—mass [g/m^2^]; *** δ—Thickness [mm]; **** Pv—Vapor permeability [%]; ***** Pa—Air permeability [L/m^2^/s].

**Table 2:** Electrical properties of the electrodes before and after treatments with artificial (alkaline/acid) perspirations.

Sample No.	PVDF	GO	Ni	Cu_1_	Cu_2_	Rs_1_ * (Ω)	Rs_2_ ** (Ω)	T *** (° C)	Conductive Effect
T1	x	x	-	-	-	10^12^	10^9^	19.2	antistatic
T2	x	-	x	-	-	10^3^	10^9^	19.1	antistatic
T3	x	-	-	x	-	10^10^	10^9^	19.1	antistatic
T4	x	-	-	-	x	10^9^	10^9^	19.4	antistatic

* Rs_1_—surface resistance before treatments in artificial perspirations; ** Rs_2_—surface resistance after treatments in artificial perspirations; *** T—the temperature at the moment of Rs_2_ investigation.

**Table 3:** Surface morphology-SEM.

Sample No.	Initial	Acid Perspiration Treatment	Alkaline Perspiration Treatment
1	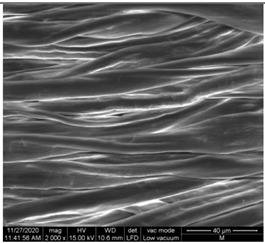	-	-
2	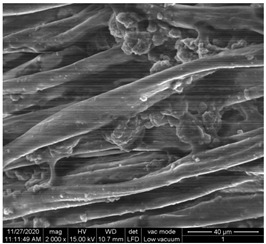	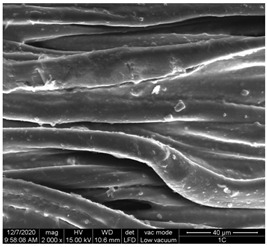	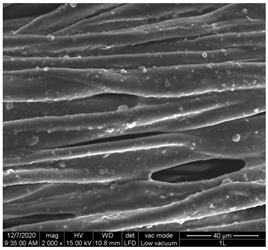
3	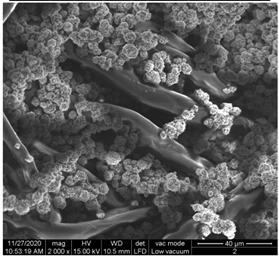	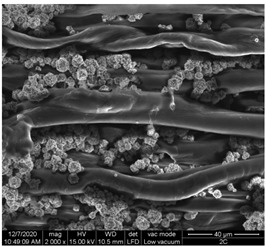	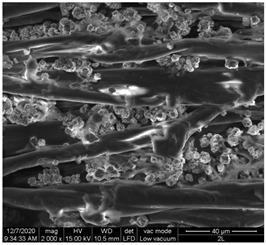
4	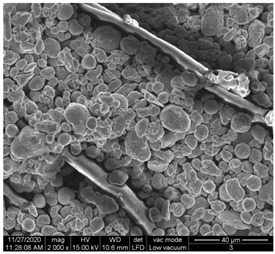	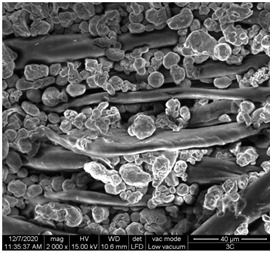	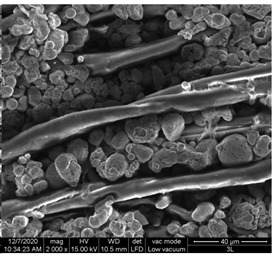
5	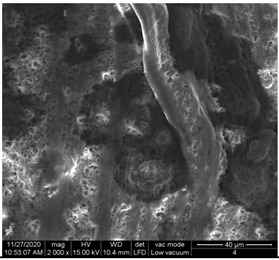	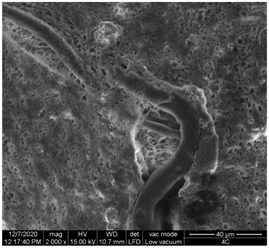	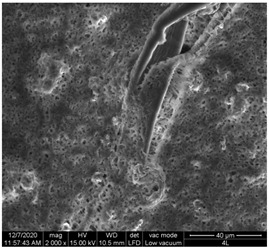

**Table 4:** Surface topography-optical microscopy and contact angle analysis.

Sample No.	Initial	After Coating	Contact Angle View	Contact Angle Value [°]
1	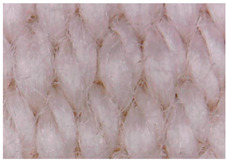	-	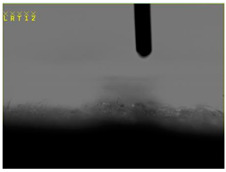	0
2	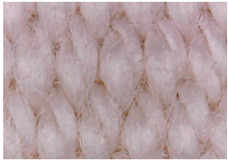	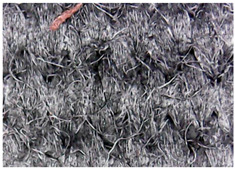	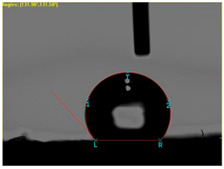	131.5
3	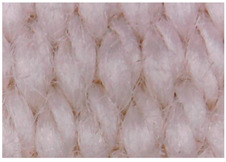	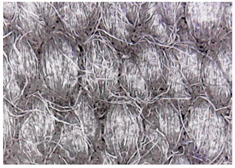	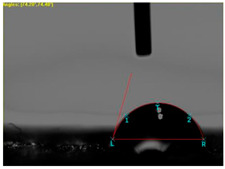	74.2
4	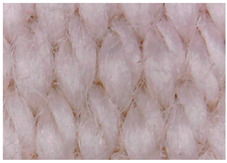	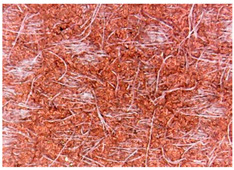	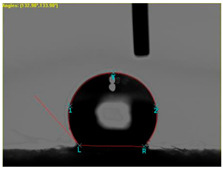	132.9
5	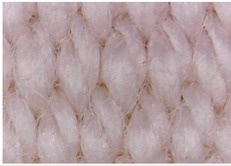	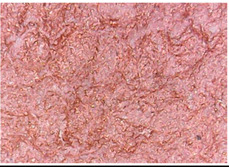	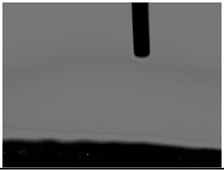	0

**Table 5:** Chemical composition (% wt).

Sample No.	C	O	F	Ni	Na	P	Cu	Si	Fe
**1**	41	59							
**2**	39	34	25						2
**2a**	32	35	32						
**2b**	33	32	35						
**3**	16	19	22	43					
**3a**	25	33	15	27					
**3b**	26	34	21	18	2				
**4**	9	15	10				66		
**4a**	15	30	9			3	43		
**4b**	15	26	12			3	40	3	
**5**	18	6	71				5		
**5a**	17	9	71			2	2		
**5b**	15	10	70			2	3		

**Table 6:** Electrode materials for sensors.

No.	Electrode Material	Rs	C	Fabrication Technique	Sensor Type	Reference
1	PVDF membrane-based nanofibers	-	-	Electrospinning	Potentiometer	[1]
2	PVDF-MWCNT	-	-	Electrospinning	Piezoelectric sensor	[2]
3	ZnO/PVDF nanofiber membrane	-	-	Electrospinning	Pressure sensor	[3]
4	PVDF/Graphene Membrane	-	-	Electrospinning	Humidity sensor	[4]
5	-	-	-	-	-	[5]
6	-	-	-	-	-	[6]
7	PVDF-based Ni membrane	10^3^ Ω	-	Coating (scrapping)	-	Actual work
8	Graphene	350/2100 Ω	-	CVD	Strain sensor	[7]
9	Graphene oxide reduced	10^3^ Ω	-			[8]
10	Graphene/Ni		-	CVD		[8]
11	Ag fabric	465 kΩ	-	Intarsia knitting	Electrode for transcutaneous electrical nerve stimulation	[36]
12	Ag printed fabric	194–953 Ω	-	Digital printing with inks-based Ag	Electrode for muscular electrostimulation	[37]
13	Ag nonwoven	10.22 Ω	-	Nonwoven	Electrode for electrostimulation	[38]
14	AgNO_3_ coated fabric	19 Ω	-	Knit coated	Electrode for electrotherapy	[39]
15	Ag/PA knitted	426 MΩ	-	Knitted electrodes based on silver-plated polyamide (Ag/PA) yarns	Electrode for biomedical monitoring	[40,41]
16	Woven-based conductive core-spun yarns	-	-	Woven electrode	Electronic Fabric Artificial Skin	[42,43,44]
17	Fiber–Bragg Gratings-based electrode	-	-	Woven electrodes	Pulse/Temperature Monitoring	[45]
18	Woven-based conductive yarns	-	-	Woven structure	Electrodes for triboelectric nanogenerator	[46]
19	Energy harvesting insole-based PVDF nanofibers	-	-	Nonwoven	Electrodes for triboelectric nanogenerator	[47]
20	PEDOT: PSS coated fabric	-	-	Coating	Electrodes for triboelectric nanogenerator	[48]
21	Fiber-based hybrid nanogenerator (FBHNG)	-	-	FBHNG-based piezoelectric nanogenerator (PENG) and triboelectric nanogenerator (TENG)	Electrodes for energy harvesting	[49]

## Data Availability

Not applicable.

## Supplementary Materials

The following supporting information can be downloaded at: https://www.mdpi.com/article/10.3390/ma15207286/s1, Table S1: Surface topography-optical microscopy and contact angle analysis for samples 6–14.
