# Supplementary material for: Conductive Membranes Based on Cotton Fabric Coated with Polymers for Electrode Applications
Source: Materials (Basel). 2022 Oct 18;15(20):7286. doi: 10.3390/ma15207286 (PMC9611807; doi:10.3390/ma15207286)
Supplement: Supplementary file 1 [file materials-15-07286-s001.zip › materials-1854753-supplementary.pdf]

Table S1. Surface topography-optical microscopy and contact angle analysis for samples 6-14

Sample no.	Initial	After Coating	Contact angle View	Contact angle value [°]
**6**	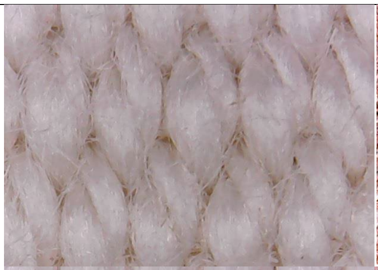	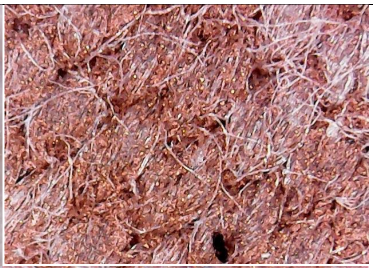	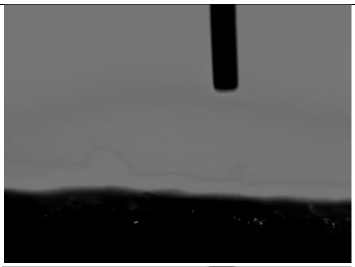	0
**7**	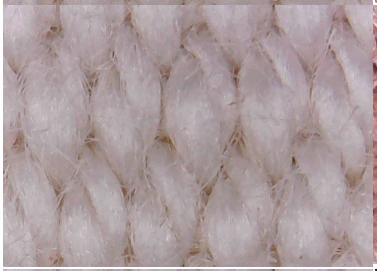	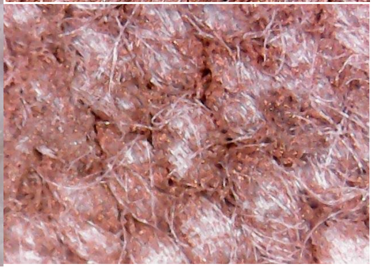	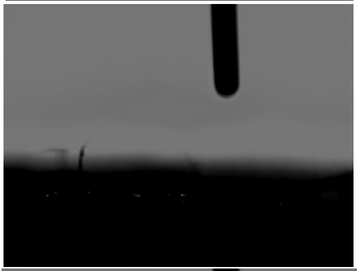	0
**8**	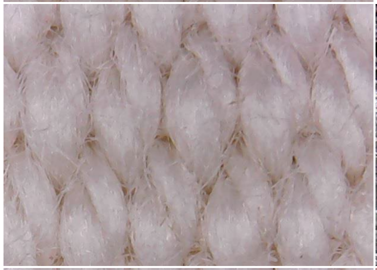	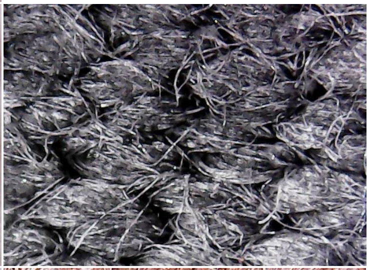	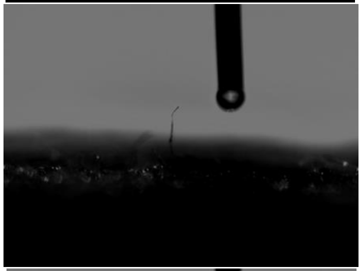	0
**9**	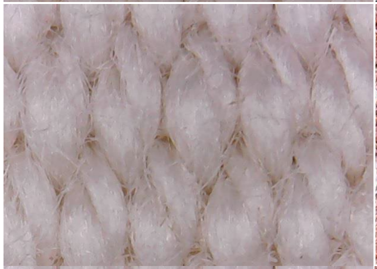	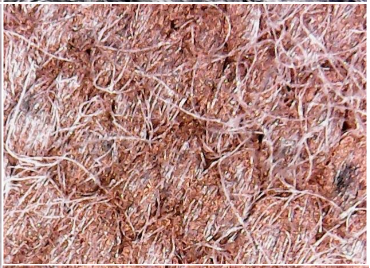	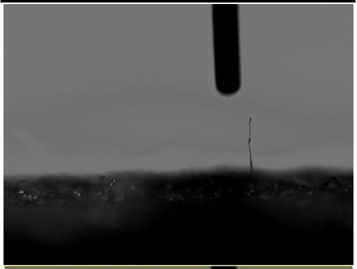	0
**10**	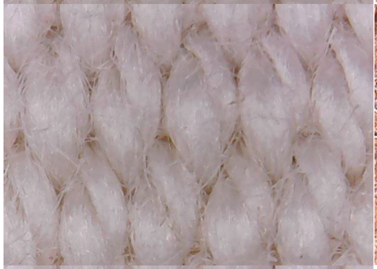	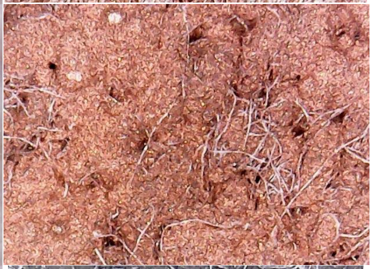	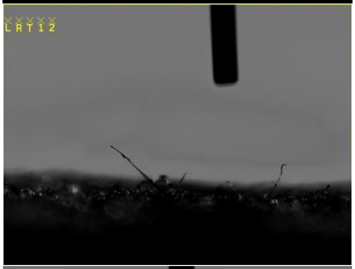	0
**11**	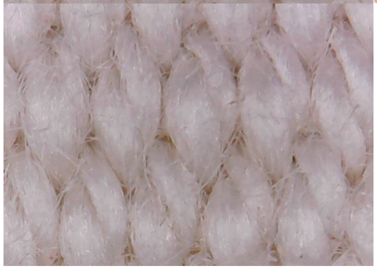	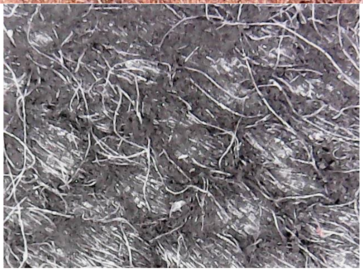	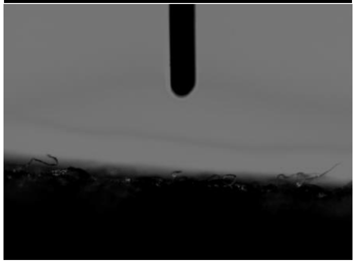	0

**12**	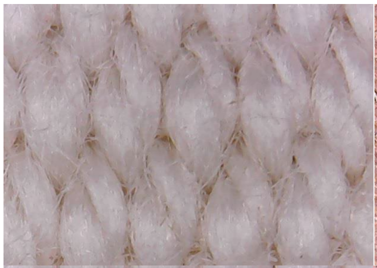	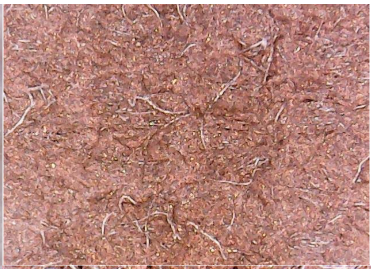	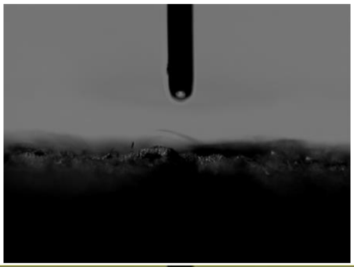	0
**13**	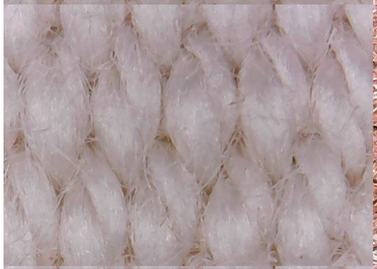	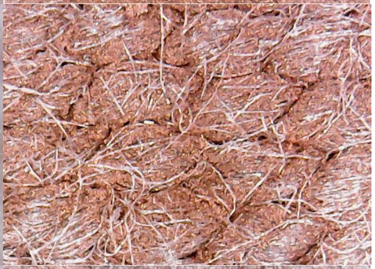	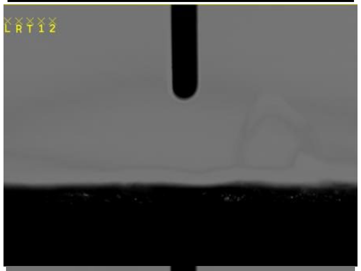	0
**14**	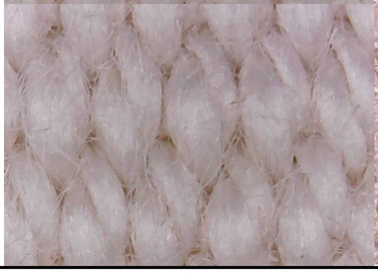	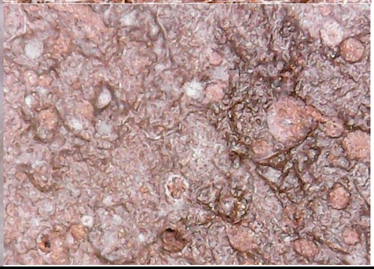	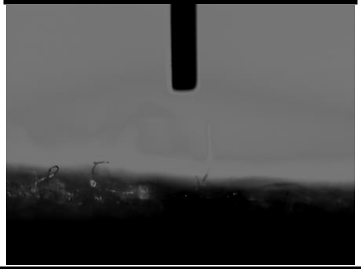	0
